# The Editor’s Choice Articles—Section “Cells of the Cardiovascular System” 2020–2021

**DOI:** 10.3390/cells11142173

**Published:** 2022-07-11

**Authors:** Kay-Dietrich Wagner

**Affiliations:** CNRS, INSERM, iBV, Université Côte d’Azur, 06107 Nice, France; kwagner@unice.fr

*Cells* is experiencing a rapid increase in attractiveness and impact. This is reflected in our recently established section “*Cells of the Cardiovascular System*”. For example, in 2019, four papers in the section were published in *Cells*, and this number increased to 79 in 2021. This is not only increasing the workload for editors and reviewers, but allows us to highlight the most exiting research and papers which are particularly interesting and important in the field on a dedicated website (https://www.mdpi.com/journal/cells/editors_choice, accessed on 10 July 2022). Nevertheless, all other published papers in our section are highly valuable for the scientific community. Non-inclusion in the editor’s choice does not mean that they are esteemed of lower importance. The selection is based solely on the editor’s personal choice. Overall, for a period from 2020 to 2021, I selected three original publications and three reviews. Here, I will summarize my view of the most interesting aspects of the selected papers.

Peroxisome proliferator-activated receptors (PPARs) are ligand-activated transcription factors with important roles in cardiovascular and metabolic diseases and cancer [1,2,3,4,5]. Fatty acids and fatty acid derivatives act as natural ligands for PPARs. Synthetic PPARα and PPARγ agonists have some clinical efficacy in reducing cardiovascular disease, which is unfortunately limited [1,2]. Thus, the identification of endogenous ligands, which might lead to the development of more potent and selective PPAR modulators for the treatment of cardiovascular disease, is of fundamental interest. Most studies in the field have used human cell lines or rodent aorta as a model. The group of D. Bishop-Bailey performed lipidomic profiling of porcine tissue explants to identify endogenous PPAR ligands [6]. As the pig cardiovascular system is close to the human situation, the model is highly relevant. The authors compared oxylipins from aorta, coronary artery, pulmonary artery, and perivascular adipose tissues. They identified the coronary artery as a major source of CYP450-derived epoxy fatty acids. These coronary artery-derived oxylipins, which have vasodilator and anti-inflammatory properties, are of interest for nutritional, lifestyle, and pharmacological interventions for prevention and treatment of ischemic heart disease.

Atrial fibrillation (AF) is another cardiovascular disease affecting a significant number of people. Blood clotting in the non-contracting atria with the risk of stroke and a general increase in mortality are the major complications of AF [7]. Although AF can be easily diagnosed by electrocardiogram in active episodes, management of intermittent AF requires additional reliable biomarkers. As mitochondrial damage is involved in AF, Wiersma and colleagues investigated whether cell-free mitochondrial DNA might be a suitable blood marker for AF. They describe that the level of mitochondrial DNA in serum is associated with AF stage and is especially elevated in males with paroxysmal AF. In addition, the level of mitochondrial DNA in blood samples is associated with recurrence of AF in patients with paroxysmal AF undergoing AF treatment [8]. These findings might have important implications for diagnosis and management of patients with atrial fibrillation and should stimulate larger clinical trials on the subject. 

To make the next step forward in innovative treatment of AF, the same groups of authors (N.M.S. de Groot and B.J.J.M. Brundel groups) performed a small pilot study of dietary supplementation with L-glutamine in patients with AF [9]. Glutamine has been shown before to induce heat-shock protein (HSP) expression [10], and induction of HSPs protected against atrial fibrillation [11]. Thus, the rationale of the study is logical and straightforward. HSP27 levels in blood samples decreased after 3 months of L-glutamine supplementation and returned to normal after 6 months, while HSP70 levels decreased after 3 months and remained low. Unfortunately, the study did not evaluate cardiac HSP levels. Further prospective clinical trials in AF patients should characterize the effects of L-glutamine supplementation on AF clinical parameters.

In the same Special Issue of *Cells* “The Role of PPARs in Disease” as the paper of D. Bishop-Bailey, the group of W. Wahli reviewed the implication of peroxisome proliferator activated receptors in non-alcoholic fatty liver disease (NAFLD) [12]. NAFLD is characterized by lipid accumulation in hepatocytes and inflammatory response, which might ultimately lead to liver fibrosis and cirrhosis. The topic is highly important as NAFLD is the most common liver disease and its prevalence is increasing rapidly alongside the number of obese subjects [13,14,15]. The authors review in detail the etiology, pathophysiology, progression, and current therapeutic strategies for NAFLD. In addition, they provide a comprehensive review of PPARs in NAFLD, of PPAR agonists in clinical use and novel agonists and their potential for the treatment of non-alcoholic fatty liver disease.

Another organ in which crosstalk between immune, vascular, and parenchymal cells in pathophysiology is increasingly recognized, is the lung. Hu and colleagues reviewed perivascular inflammation in pulmonary arterial hypertension (PAH) [16]. As the inflammatory response in PAH correlates with vascular remodeling, hemodynamic parameters, and clinical outcome [17], modifying this inflammatory response and vascular remodeling might offer novel therapeutic approaches. The authors summarize in their review the current knowledge on cytokines, inflammatory mediators, and immune cell types and their effects on vascular cells and consequence for PAH as well as the vascular remodeling-induced phenotypic changes which affect in turn immune cells. In their conclusion, they mention that it took 4500 years from first attempts of immunotherapy for cancer until the development of checkpoint inhibitors. We hope that thanks to the high-quality papers and informative reviews published in *Cells*, the process of novel therapeutic developments might reasonably speed up.

As a pre-requisite to modifying vascular phenotype and response, knowledge on the differences in distinct vascular beds is required. Hennigs et al. reviewed in detail the heterogeneity of vascular endothelial cells. They first introduce the general characteristics of the endothelium and physiological endothelial heterogeneity, which is a timely introduction to the topic for scientists working in the field. Afterwards, modifications of the endothelium in response to stress, i.e., inflammation, ischemia, and cancer, are discussed, and finally the different strategies to target distinct endothelial cell populations are reviewed. This provides an excellent overview and guidance for researchers aiming at selective endothelial cell modifications.
